# Supplementary material for: Linear mitochondrial DNA is rapidly degraded by components of the replication machinery
Source: Nat Commun. 2018 Apr 30;9:1727. doi: 10.1038/s41467-018-04131-w (PMC5928156; doi:10.1038/s41467-018-04131-w)
Supplement: Supplementary file 1 — Supplementary Information [file 41467_2018_4131_MOESM1_ESM.pdf]

SUPPLEMENTARY INFORMATION

Linear mitochondrial DNA is rapidly degraded by components of the replication machinery

Peeva et al.

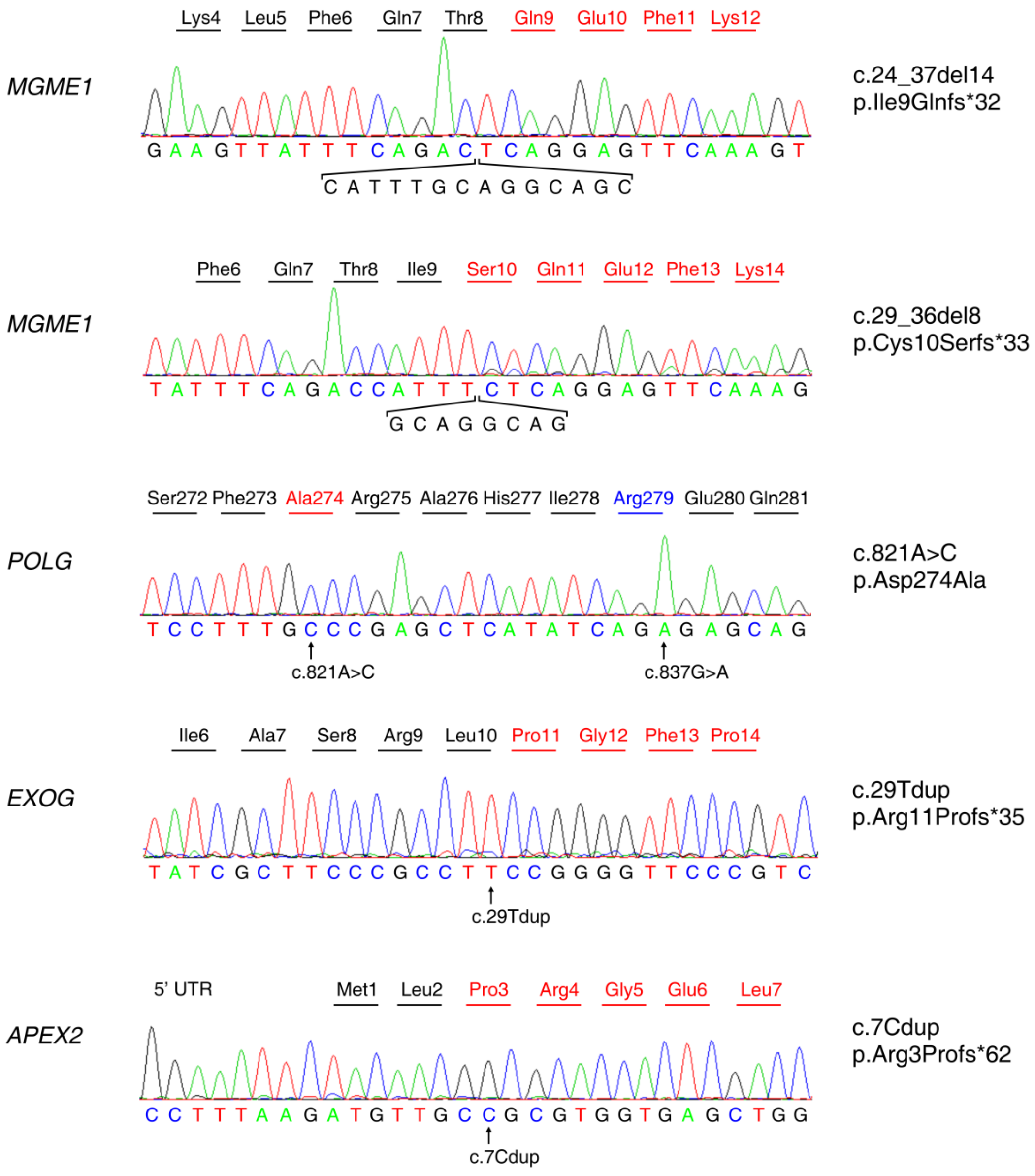

Supplementary Figure 1 Knockout and knock-in cell lines generated using CRISPR–Cas9. Sequencing chromatograms confirming the knockout of *MGME1*, *EXOG*, *APEX2* and the p.D274A knock-in of *POLG* in different mitoEagI HEK 293 cells. Red amino acid codes indicate missense changes. In case of the *POLG* knock-in, blue amino acid code refers to a silent nucleotide change that was additionally introduced in order to disrupt a protospacer adjacent motif (PAM).

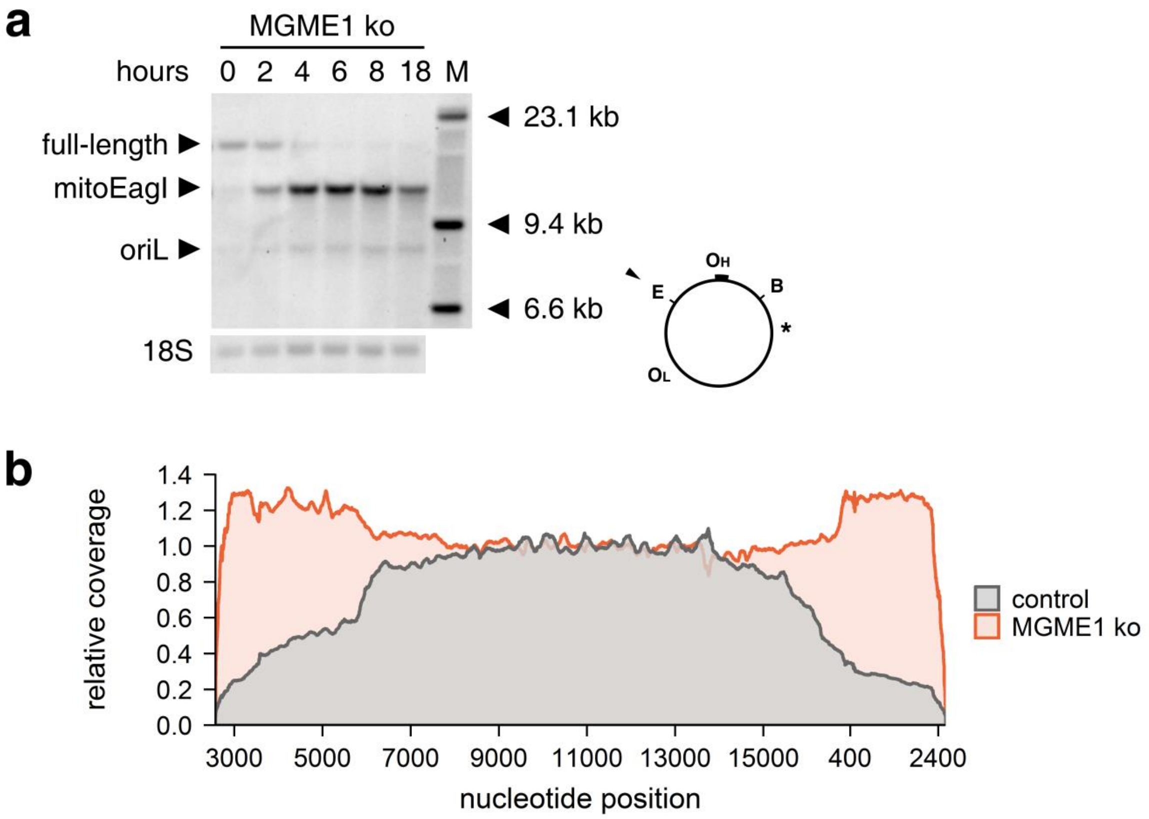

Supplementary Figure 2 Degradation of mtDNA in the mitoEagI-expressing MGME1 p.C10Sfs*33 knockout cell line. **a** Southern blot showing the degradation of mtDNA within the first 18 hours of induced expression of mitoEagI (E) in MGME1 knockout cells. BamHI endonuclease-linearized DNA (B) was labeled with a mitochondrial probe represented by an asterisk as well as a probe specific for nuclear 18S ribosomal DNA (‘18S’). **b** Coverage ratios throughout the mitochondrial genome as determined by ultra-deep sequencing of mtDNA from cells 6 hours after induced mitoEagI expression and normalized to values in non-induced cells.

Supplementary Figure 3

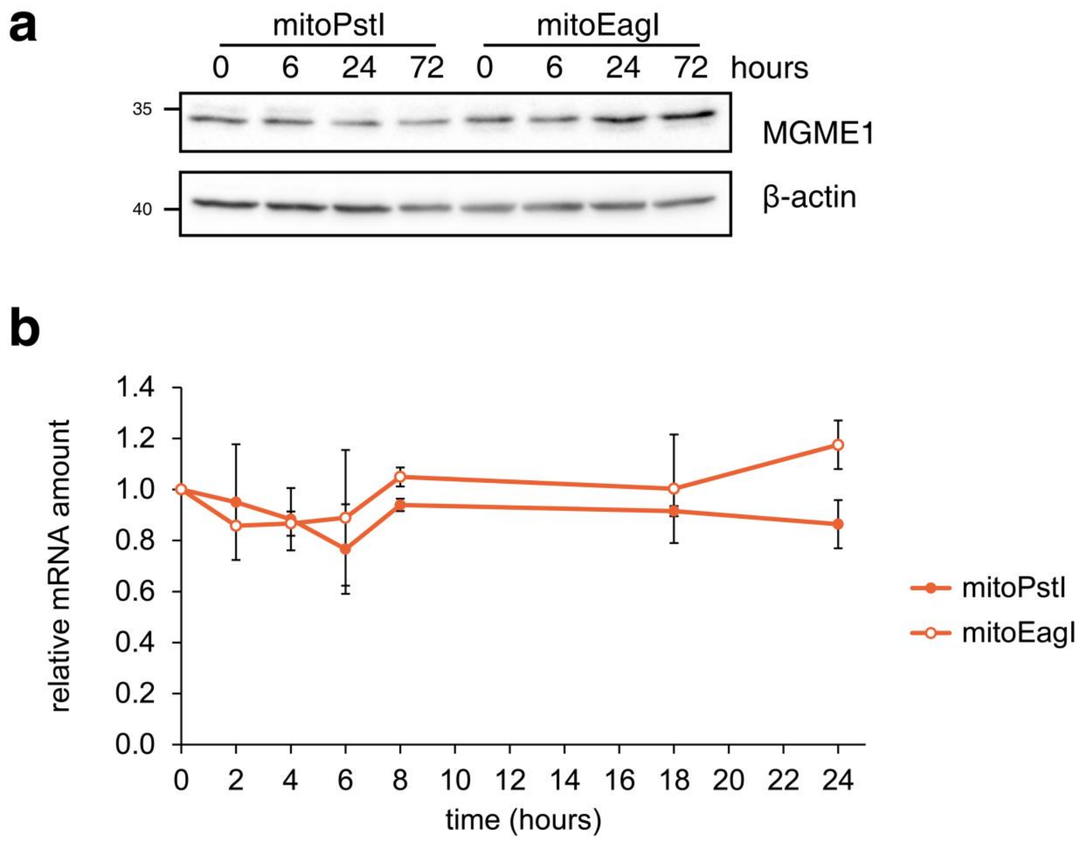

Supplementary Figure 3 MGME1 mRNA and protein levels are not altered upon induction of mtDNA double-strand breaks. **a** Western blot demonstrating that MGME1 protein amounts remain unaltered in control cells after induced mitoPstI or mitoEagI expression. Protein molecular weight marker band sizes in kDa are indicated on the side. **b** Reverse-transcriptase quantitative PCR analysis showing no changes in expression of *MGME1* at the mRNA level upon induced mitoPstI or mitoEagI expression. Values were normalized to values before induction (‘0 hour’). Error bars represent standard error of the mean (SEM) from two independent experiments.

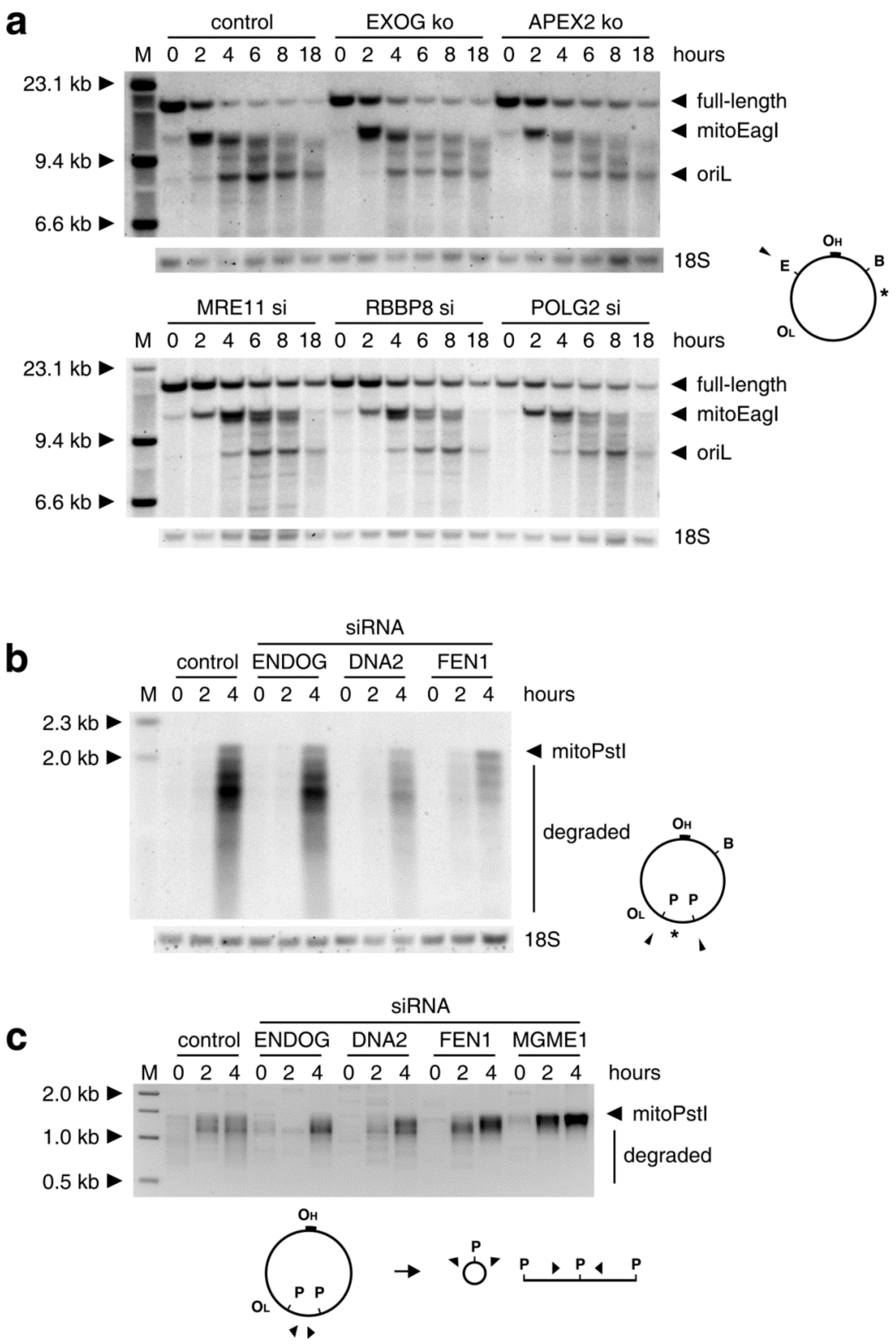

Supplementary Figure 4 Rapid degradation of mtDNA is not affected by inactivation of EXOG, APEX2, ENDOG, DNA2, FEN1, MRE11, RBBP8, or POLG2. **a** Southern blot demonstrating comparable dynamics of mtDNA degradation in control, *EXOG* knockout and *APEX2* knockout cells as well as in *MRE11*, *RBBP8*, and *POLG2* siRNA knock-down cells within the first 18 hours of induced mitoEagI (E) expression. DNA was cleaved by BamHI (B) and labeled with a probe located at position 12602–12690 indicated by an asterisk. Note: No POLG2- specific antibodies were available to validate the steady-state protein level in the siRNA experiment, however, the low *POLG2* mRNA level and the decreased mtDNA copy number in *POLG2* siRNA knock-down cells confirm the efficiency of the knock down (Supplementary Table 1). **b** Southern blot showing the lack of effects on

the degredation of the 2.1-kb fragment in mitoPstI-expressing (P) cells upon *ENDOG*, *DNA2* or *FEN1* knock down. Asterisk indicates the probe located at position 7771–8009. **c** PCR amplification of breakpoints related to the ends of the 2.1-kb mtDNA fragment in mitoPstI-expressing cells. Amplification primers MT8282F and MT7682R (Supplementary Table 4) are shown in the scheme as arrowheads. Mainly breakpoints originating from degraded ends are detectable in control, *ENDOG* siRNA, *DNA2* siRNA and *FEN1* siRNA cells. Breakpoints observed in *MGME1* knock-down cells mainly correspond to non-degraded ends.

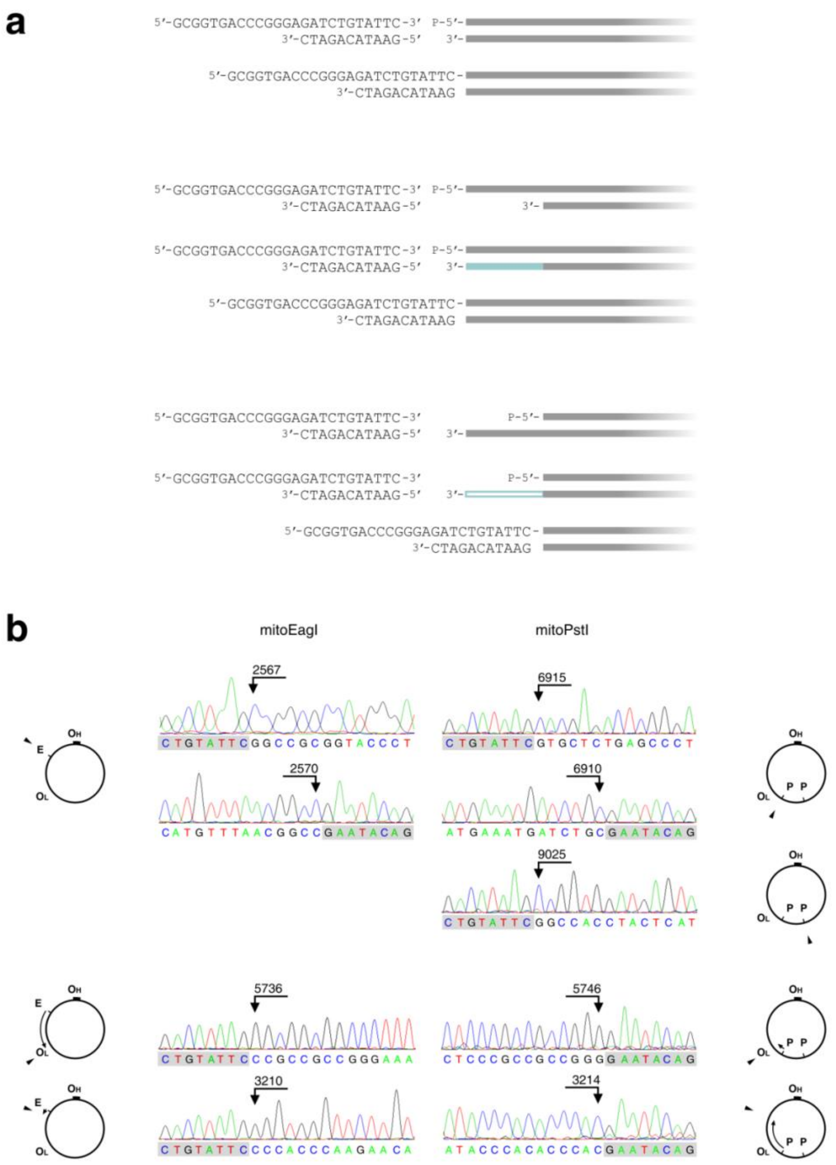

Supplementary Figure 5 Linker-ligated mtDNA ends. **a** Principle of detecting mtDNA ends by ligation with an asymmetric double-stranded linker. Nucleotide sequence of the linker is shown. Gray lines represent strands of linear mtDNA species. Blunt mtDNA ends can be directly ligated with the linker (upper part). 5' overhangs are only ligatable to the linker if they are first filled in by T4 polymerase treatment (middle part, colored line). 3' overhangs are removed by T4 polymerase treatment (lower part, contoured line). **b** Representative free mtDNA ends in mitoEagI-expressing and mitoPstI-expressing HEK 293 cells. Ends of linear mtDNA molecules were detected by Sanger sequencing of single-molecule ligation-mediated PCR amplicons in mitoEagI-expressing (left panels) and mitoPstI-expressing (right panels) HEK 293 cells 6 hours after induction. The upper set of chromatograms shows non-degraded mtRE-generated ends. The lower set of chromatograms displays partially degraded ends distal to cutting sites. Localization of restriction endonuclease recognition sites on the mitochondrial genome are indicated in the schemes on the sides (E, mitoEagI; P, mitoPstI). Arched arrows indicate directions of degradation that result in specific ends. Gray shadings under the chromatograms indicate the end of the linker sequence, arrows point to determined end positions.

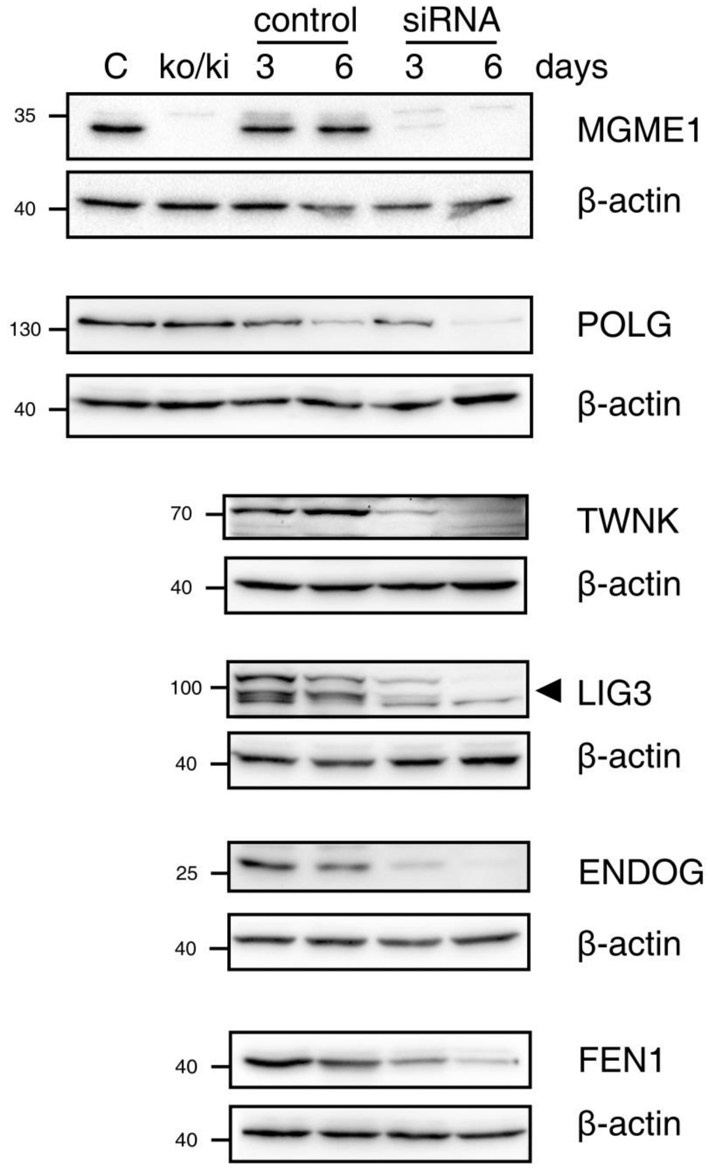

Supplementary Figure 6 Western blot analysis of protein depletion in siRNA-treated HEK 293 cells. Whole cell protein extracts of cells treated with various siRNAs for 3 or 6 days are shown along with mock-treated control cells at the same time points. β-actin was used as loading control. C, untreated control mitoEagI cells; 'ko/ki', MGME1 knockout or POLG knock-in mitoEagI cells. All other lanes represent mitoPstI cells. The arrowhead in the LIG3 blot marks the mitochondrial isoform. Protein molecular weight marker band sizes in kDa are indicated on the side.

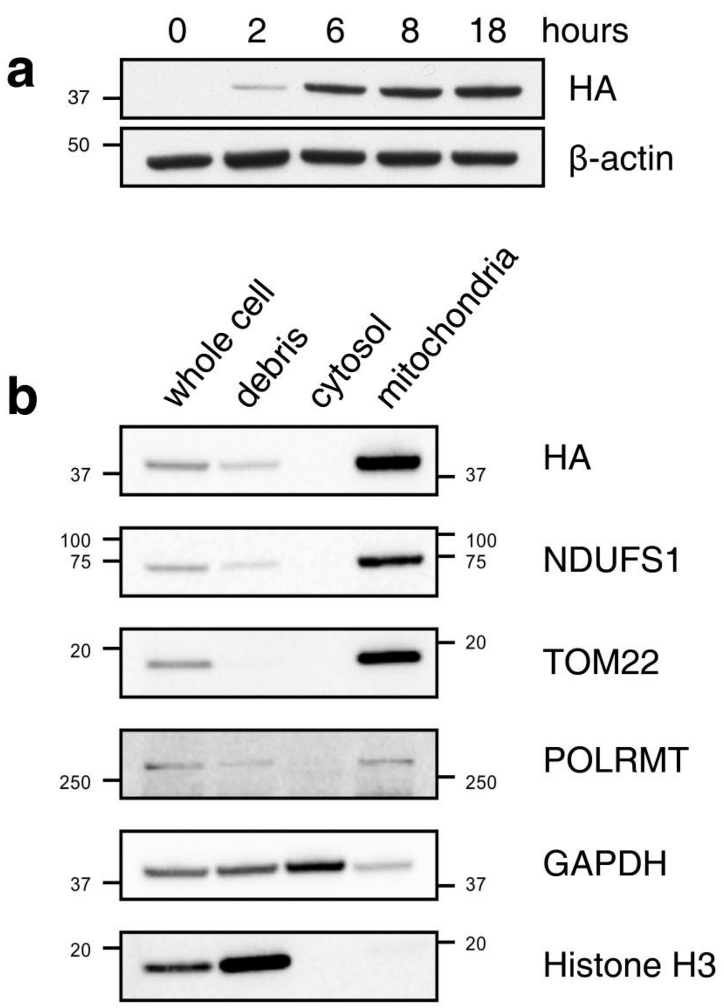

Supplementary Figure 7 Inducible expression and mitochondrial localization of mitoEagI by Western blot analysis. **a** Whole cell protein extracts of non-induced (‘0 hour’) and induced mitoEagI-expressing HEK 293 cells. β-actin was used as loading control. Protein molecular weight marker band sizes in kDa are indicated on the side. **b** Subcellular fractions by differencial centrifuging. Note that the HA-tagged mitoEagI is highly enriched in the mitochondrial fraction, similar to other mitochondrial proteins NDUFS1, TOM22, and POLRMT.

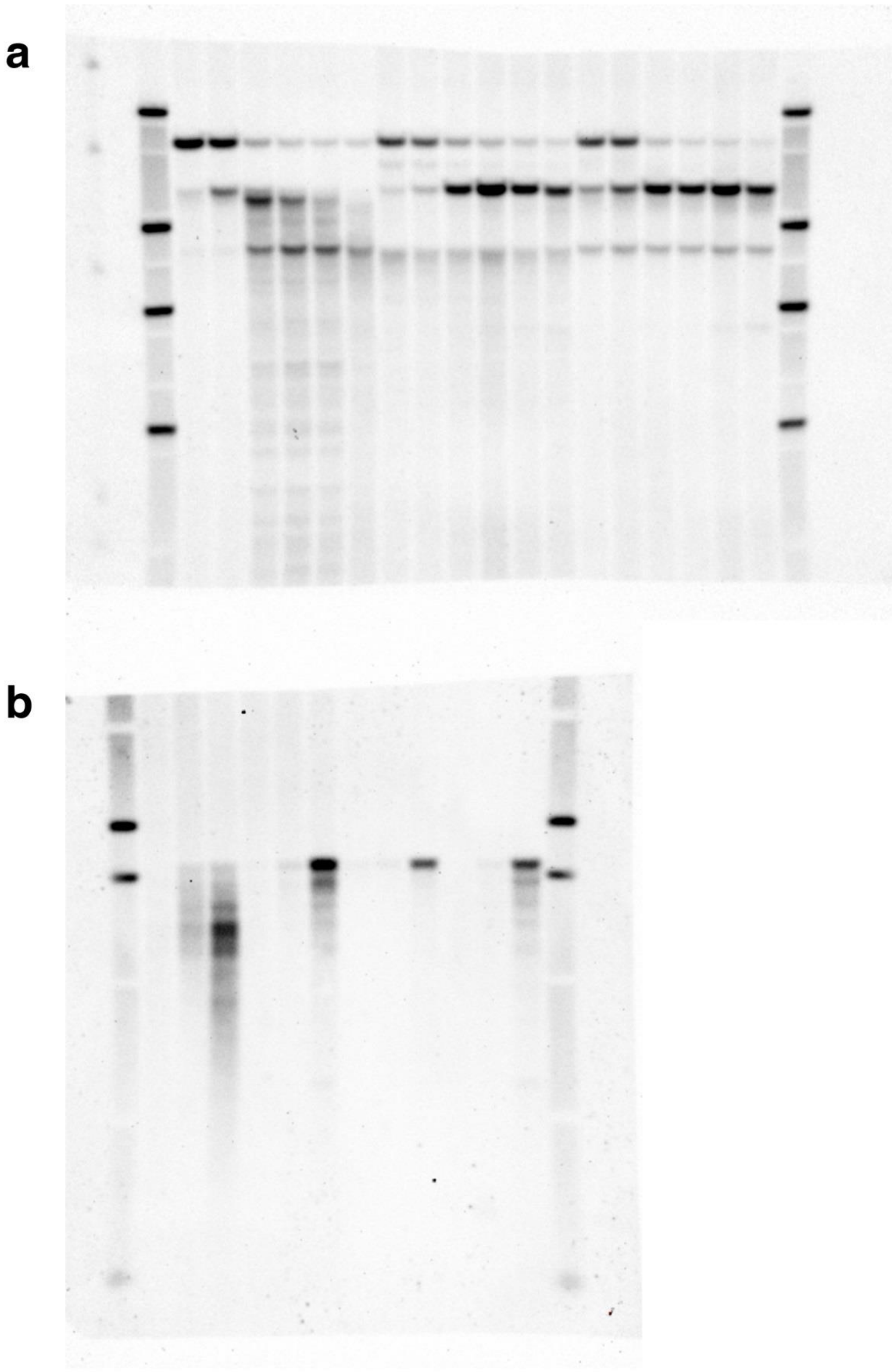

Supplementary Figure 8 Uncropped scans of the most important blots. **a** Uncropped scan used in Fig. 1a. **b** Uncropped scan used in Fig. 2a.

Supplementary Table 1

Knock-down of genes by siRNA in mitoPstI or mitoEagI HEK 293 cells

Gene	siRNA	Knock-down efficiency[a]	mtDNA copy number[c]
MGME1	CCACGAAGCCUUGGAAAGCAUACUU	99%	1074 ± 389
POLG	GCAAGGAAGUCACAGUGGAAGAAGU<br>GGCACAGAGUCAGAAAUGUUCAAUA<br>GCUUCUGCAUCAGCAUCCAUGACGA	40%	640 ± 32
TWNK	GCCGCUUUCCAGACCUCAAUCGUAU	90%	230 ± 66
ENDOG	GGAACAACCUGGAGAAAUATT	97%	1376 ± 75
DNA2	GCCUGCAUUCUAAAGGGUUUGAAUA	80%	1456 ± 297
FEN1	CAUCAAGCCCGUGUAUGUCUUUGAU	82%	1163 ± 249
LIG3	GCCAAUGACCUUAAAUGCAUCAUCA	94%	2139 ± 214
MRE11[b]	ACAUGUUGGUUUGCUGCGUAUUAAA<br>UCAUGGAGGAUAUUGUUCUAGCUAA<br>UUAGCUAGAACAAUAUCCUCCAUGA	81%	2073 ± 80
RBBP8[b]	GGGUCUGAAGUGAACAAGAUCAUUA<br>ACGUCAGCCUUACAACGCAAUAUUU<br>GACGUUAUUCCAGAUUCACCGAUAA	86%	1040 ± 70
POLG2[b]	GACAGGUUUGUCAAGGGCUAUUUAA<br>CCACAAUGAAGGAAAUGAUGCAUAU<br>GAGAAGACUGAAGCUUCGUUAGUAU	75%	619 ± 15

[a] mRNA amounts as determined by reverse transcriptase quantitative PCR.
[b] Knock-down performed in mitoEagI HEK 293 cells.
[c] mtDNA copy number in control mitoPstI HEK 293 cells was 1493 ± 361 and in mitoEagI cells 1118 ± 195.

Supplementary Table 2

mtDNA breakpoints in mitoPstI-expressing HEK 293 cells in the vicinity of the cutting sites

Sample	Start	Stop	Count	Repeat length[a]	Repeat sequence[b]	PrimerF	PrimerR
control	8373	6954		1 / 1		8282F	7682R
	8398	7332		1 / 1			
	8530	7382		2 / 2			
	8563	6954		1 / 1			
	8563	7078		0 / 0			
	8564	7089		3 / 10	GCTTCATTCA		
	8564	7160		0 / 0			
	8611	7493		1 / 4			
	8734	6915		0 / 6	TGATCT		
	8860	7072		0 / 0			
	8864	7073		1 / 5	ATAGG		
	8882	7500		1 / 1			
	8884	6922		0 / 5	GCTCT		
	8899	7078		0 / 0			
	8899	7596		0 / 4			
	8904	6953		0 / 0			
	8944	6922		0 / 4			
	8971	7269		0 / 8	CTCATTCA		
	8977	7111		0 / 8	CTCATTCA		
	8997	6921		1 / 1			
	8998	7009		2 / 5	CGTAC		
	9005	7130		0 / 6	TACGCC		
	9006	7126		1 / 6	TACGCC		
	6517	9025		0 / 4		6377F	9795R
	6452	9025		0 / 0			
	6574	9026		1 / 1			
	6577	9025		0 / 0			
	6623	9025		0 / 4			
	6623	9141		1 / 1			
	6625	9025	2	0 / 4			
	6625	9153		0 / 0			
	6670	9025		2 / 2			
	6827	9025		0 / 0			
	6847	9049		0 / 4			
	6855	9025		0 / 0			
	6893	9025		0 / 0			
MGME1 si	8373	6954		1 / 1		8282F	7682R
	8564	7130		0 / 4			
	8610	7211		1 / 4			
	8734	6915		0 / 6	TGACTC		
	8769	7126		1 / 5	AACCT		
	8771	7129		0 / 5	AACCT		
	8851	7126		0 / 0			
	8863	6915		0 / 6	GCAGTG		
	8863	7078		2 / 6	AGGCTT		
	8882	6915	2	0 / 5	GCTCT		

continued

Sample	Start	Stop	Count	Repeat length[a]	Repeat sequence[b]	PrimerF	PrimerR
MGME1 si	8899	7026		0 / 4		8282F	7682R
	8900	6915		0 / 0			
	8901	6977		0 / 5	CGTAC		
	8902	6916		2 / 2			
	8904	6914		1 / 1			
	8904	6915		0 / 0			
	8904	7125		0 / 6	CCCTAG		
	8907	6915		0 / 0			
	8927	6964		0 / 4			
	8971	6915		0 / 0			
	8994	7007		0 / 0			
	8995	6915		0 / 0			
	8995	6916		1 / 1			
	8998	7077		0 / 0			
	8998	7208		1 / 1			
	8999	7157		1 / 1			
	9005	7379		3 / 4			
	9017	7129		1 / 1			
	6574	9026		1 / 1		6377F	9795R
	6625	9025		0 / 4			
	6731	9025		0 / 0			
	6879	9025		2 / 5	GCCAC		
	6893	9025	2	0 / 0			
	6909	9025		0 / 6	CTGCAG		
	6910	9025	11	0 / 6	CTGCAG		
	6910	9041		3 / 4			
	6910	9029		1 / 6	CTGCAG		
	6911	9036		2 / 4			
TWNK si	6625	9025		0 / 4		6377F	9795R
	6625	9052		0 / 4			
	6750	9025		0 / 4			
	6825	9026		1 / 5	CACCT		
	6858	9025		2 / 2			
	6882	9025		0 / 5	GCCAC		
	6910	9025	7	0 / 6	CTGCAG		

Breakpoints were determined by sequencing single-molecule PCR products that were amplified using the indicated primers. 'Start' and 'Stop' indicate the last and first retained nucleotide positions, respectively. Count values are indicated if breakpoints were detected more than once. PrimerF, forward primer used for single-molecule PCR amplification; PrimerR, reverse primer.

[a] Direct repeat lengths are indicated. First number stands for perfect direct repeat exactly at the breakpoint. Second number represents perfect direct repeat in the vicinity of the breakpoint. Underlined numbers indicate repeat length of at least 5 base pairs.

[b] Sequences of perfect direct repeats are shown if their lengths are of at least 5 nucleotides.

Supplementary Table 3

mtDNA breakpoints in patients suffering from mtDNA maintenance disorders

Deficiency	Start	Stop	Count	Repeat length[a]	Repeat sequence[b]	PrimerF	PrimerR
MGME1	1109	15975		1 / 4		1056F	1144R
	1171	16038	2	3 / 3			
	1275	3224		2 / 2			
	1726	355		8 / 8	GCCAAACC		
	1900	16002		12 / 12	CCAAAGCTAAGA		
	1948	345		0 / 4			
	3257	16071		2 / 2			
	3270	615		0 / 4			
	3301	11724		4 / 4			
	3418	13058	2	9 / 9	AAGGCCCCA		
	3474	13627		1 / 5	TCACC		
	3528	15750		1 / 4			
	3532	14351		0 / 4			
	3575	13685		3 / 5	CCCCA		
	3576	314		3 / 11	CCCCCCTCCCC		
	3578	14820		6 / 6	CCCCAT		
	3720	15946	2	0 / 4			
	6601	616		1 / 4			
	9080	15245		1 / 1			
POLG	1726	355		8 / 8	GCCAAACC	1056F	1144R
	2438	530		0 / 4			
	3577	14814		8 / 8	CCTCCCCA		
	5830	15069		0 / 0			
	5830	15573		0 / 0			
	6129	13950		0 / 0			
	6210	15158		0 / 4			
	6341	14005		11 / 11	TAGACCTAACC		
	6933	15965		0 / 5	TCTTT		
	7469	14813		4 / 5	ACCCC		
	7821	16072		1 / 6	CCCATC		
	8512	15967		3 / 3			
	8563	15239		0 / 4			
TWNK	1328	373		0 / 0		1056F	1144R
	3260	11934		1 / 1			
	3417	15661		3 / 5	CCCCA		
	3675	14622		0 / 4			
	4379	12299		0 / 0			
	5457	14652		10 / 10	CCCACACTCA		
	5744	13923	2	0 / 0			
	5786	13919		1 / 4			
	5787	13156	2	2 / 2			
	5787	13924		1 / 4			
	5787	16078		0 / 0			
	5790	13924		1 / 4			
	6175	11266		1 / 1			
	6295	13062		2 / 4			

continued

Deficiency	Start	Stop	Count	Repeat length[a]	Repeat sequence[b]	PrimerF	PrimerR
TWNK	6341	14005		11 / 11	TAGACCTAACC	1056F	1144R
	6426	13591		5 / 5	CCCTG		
	6465	12962		0 / 4			
	6625	16073		2 / 6	TCACCC		
	6781	10131		0 / 4			
	6870	14308		4 / 4			
	6929	13564		10 / 10	CTGAGCCCTA		
	7494	15530		10 / 10	AGCCAACCCC		
	7817	16069		0 / 6	CCCATC		
	8663	15388		4 / 4			
	8855	15736		2 / 5	CGCAG		
	3256	15970		1 / 4		3137F	45R
	3263	12300		0 / 4			
	3263	13923		0 / 0			
	3531	15969		0 / 0			
	4356	13925		2 / 4			
	4356	14158		4 / 4			
	4376	13923	3	0 / 5	ATTCT		
	4989	13924		1 / 4			
	5337	13923		0 / 5	TCTAC		
	5580	15664		1 / 1			
	5775	14425		0 / 0			
	5787	16072		1 / 1			
	5864	15960		3 / 3			
	5893	13452		5 / 7	CCTCACC		
	5898	14818		6 / 6	CACCCC		
	7183	13055		2 / 5	CCCAC		
	7568	14844		2 / 4			
	7842	15616		0 / 0			
	9923	16073		2 / 2			
	10747	15263		1 / 1			
	11237	16072		1 / 5	ACTCA		

Breakpoints were determined by sequencing single-molecule PCR products that were amplified using the indicated primers. Primers 1056F and 1144R were used in long-extension PCR to amplify partial mtDNA duplications. 'Start' and 'Stop' indicate the last and first retained nucleotide positions, respectively. Count values are indicated if breakpoints were detected more than once. PrimerF, forward primer used for single-molecule PCR amplification; PrimerR, reverse primer.

[a] Direct repeat lengths are indicated. First number stands for perfect direct repeat exactly at the breakpoint. Second number represents perfect direct repeat in the vicinity of the breakpoint. Underlined numbers indicate repeat length of at least 5 base pairs. Note that our previously published analyses of breakpoints in patients carrying pathogenic *MGME1* and *POLG* mutations were restricted to the first value.

[b] Sequences of perfect direct repeats are shown if their lengths are of at least 5 nucleotides.

Supplementary Table 4

DNA oligonucleotides used in the study

Primer name	Sequence	Annealing temperature (°C)[a]
Southern probes		
MT12602F	TCATCCCTGTAGCATTGTTCG	55.5
MT12690R	GAAGAACTGATTAATGTTTGGGTCT	55.5
MT7771F	AACCGTCTGAACTATCCTGC	55.0
MT8009R	CTCGATTGTCAACGTCAAGG	55.0
18SRRNAF	GTTGGTGGAGCGATTTGTCT	55.5
18SRRNAR	GGCCTCACTAAACCATCCAA	55.5
qPCR for mtDNA copy number		
MT3922F	GAACTAGTCTCAGGCTTCAACATCG	62.5
MT4036R	CTAGGAAGATTGTAGTGGTGAGGGTG	62.5
KIR835F	GCGCAAAAGCCTCCTCATT	62.5
KIR903R	CCTTCCTTGGTTTGGTGGG	62.5
Mitochondrial genome		
MT1056F	AGCTAAGACCCAAACTGGGATT	68.0
MT1144R	AGTGTTCTGGCGAGCAGTTTTG	68.0
MT3137F	GAGAAATAAGGCCTACTTCACAAAGC	68.0
MT45R	TGGAGAGCTCCCGTGAGTGGTT	68.0
MT6377F	CTTAGGGGCCATCAATTTCATCAC	68.0
MT9795R	ATGTTGAGCCGTAGATGCCGTC	68.0
MT8282F	CCCCTCTAGAGCCCACTGTA	68.0
MT7682R	GGAAAATGATTATGAGGGCGTGAT	68.0
MT14588F	CCCCCATAAATAGGAGAAGGCTTA	55.5
MT14695R	GGTTGTAGTCCGTGCGAGAA	55.5
MT8194F	CCACAGTTTCATGCCCATCGTCC	68.0
MT8387R	CGGTAGTATTTAGTTGGGGCATTTCAC	68.0
Reverse transcriptase qPCR		
MGME1_CF	ACAGCGGATGATTCTGGAAC	62.6
MGME1_CR	TCGCACTCCACTGACATCTT	62.6
POLG_CF	TGGCAGGAGCAGTTAGTGGT	62.6
POLG_CR	TGCTATCCACAGACTGCGCT	62.6
TWNK_CF	GGCTGGAAGATCAACTGGACAA	68.6
TWNK_CR	ACTGCAGGTTGTCGATGATCAC	68.6
ENDOG_CF	GGACGACACGTTCTACCTGA	57.6
ENDOG_CR	CCTGTGCAGACATAGACGTT	57.6
DNA2_CF	CATGGTGCCATACCTGTCACA	62.5

continued

Primer name	Sequence	Annealing temperature (°C)[a]
DNA2_CR	GAAGGACCGACAAGTTTCTGTC	62.5
FEN1_CF	TGTCCCAAAGGCCAGTCATC	62.5
FEN1_CR	AGAGGCATCAATGGCCACCT	62.5
PMPCA_CF	CATGTATGCTGTGTCTGCTGATAGCA	66.2
PMPCA_CR	GTGTTCTCCCTGTAAGCCGCTTCATG	66.2
MRE11_CF	GGTGATCGGCCTGTCCAGTT	62.1
MRE11_CR	AATGTTGAGGTTGCCATCTT	62.1
RBBP8_CF	ACAGGAACGAATCTTAGATG	62.1
RBBP8_CR	AGCCTGCTCTTAACCGATCT	62.1
POLG2_CF	GGACAGTGCCTTCAGGTTAG	62.1
POLG2_CR	GCCATAAGGTAGCCTCTTGT	62.1
CRISPR–Cas9		
CAS9UNIV	Phos-AACGGACTAGCCTTATTTTAACTTGCTATTTCTAGCTCTAAAAC	
POLG_SG*	GGAAAGGACGAAACACCG–CTTTGACCGAGCTCATATC–<br>–GTTTTAGAGCTAGAAATAGCAAGTTAAAATAAGG	
POLG_HDRF**	GTCCCTACTGGTGCCAGCAGCCCCACCCAGAGAGACTGGCAGGAGCAGTTAGT–<br>–GGTGGGGCACAATGTTTCCTTTGCCCGAGCTCATATCAGAGAGCAGTACCTGA–<br>–TCCAGGTAAGGTTCCTGGGGCCAACTGCCGGTTCTGGCATGG	
EXOG_SG*	GGAAAGGACGAAACACCG–GTATCGCTTCCCGCCTCCG–<br>–GTTTTAGAGCTAGAAATAGCAAGTTAAAATAAGG	
APEX2_SG*	GGAAAGGACGAAACACCG–CCCTTTAAGATGTTGCGCG–<br>–GTTTTAGAGCTAGAAATAGCAAGTTAAAATAAGG	
CAS9PLF	GAGGGCCTATTTCCCATGAT	
CAS9PLR	TTATGTAACGCGGAACTCCA	
MGME1_GF	GTTCACTTTTTTCTTCCGGCCAC	65.0
MGME1_GR	CTGATGCAGCTTTCGCTTTGATG	65.0
POLG_GF	AAATGCCACAGAGACGAAGG	66.0
POLG_GR	CAGTGGTTGTTGTGGAGTGG	66.0
EXOG_GF	ATCATATTCCCCATCCATCG	62.0
EXOG_GR	TCTGACCTTTATTCTCCCGG	62.0
APEX2_GF	CTGAACAGGAAGCAGTTCGC	65.0
APEX2_GR	CAAGGGGTGGCAAATCAGGA	65.0
Linker		
LINK25F	GCGGTGACCCGGGAGATCTGTATTC	
LINK11R	GAATACAGATC	

[a] Temperature is indicated if the oligonucleotide was used for PCR
*Gene-specific sgRNA sequences are underlined
**Introduced mismatches are underlined
